# Correction to ‘Cluster-independent marker feature identification from single-cell omics data using SEMITONES’

**DOI:** 10.1093/nar/gkad785

**Published:** 2023-10-09

*Nucleic Acids Research*, Volume 50, Issue 18, 14 October 2022, Page e107, https://doi.org/10.1093/nar/gkac639

In the originally published version of this manuscript, Panel D of Figure 3 was incorrect, and the referring text to that figure should be updated as follows.

Originally the article stated: ‘In terms of the median AUROC score, SEMITONES outperforms all alternative methods when using the suggested RBF-kernel over a multidimensional UMAP-embedding (Figure 3D, left). Similarly, using SEMITONES with a cosine similarity over a PCA-embedding results in superior or equivalent performance compared to the next-best performing methods.’

The correct statement is: ‘In terms of the median AUROC score, SEMITONES outperforms alternative clustering-independent methods across most combinations of simulations, multidimensional embeddings, and similarity metrics (Figure 3D, left). Additionally, when using the suggested RBF-kernel over a multidimensional UMAP-embedding, SEMITONES shows comparable performance to differential expression testing using MAST or Wilcoxon rank-sum testing without prior gene filtering. Of note, these differential expression testing methods were provided with the ground-truth clusters and trajectories between which differential expression was simulated, giving them an advantage over alternative methods due to prior information accessibility and using an identical marker gene definition.’

**Figure 3.**
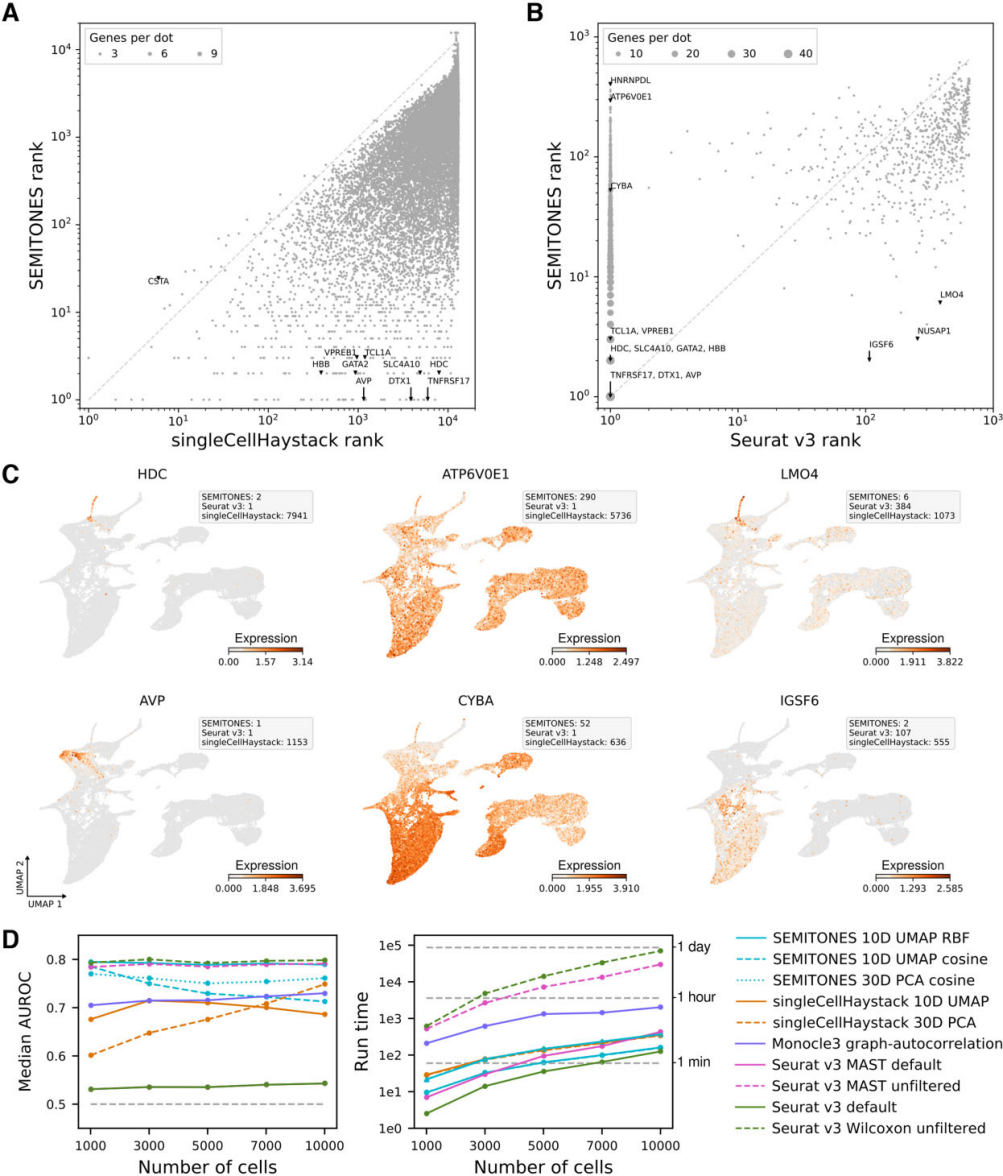
Comparison of SEMITONES to alternative marker identification methods. (**A**) Gene ranks based on SEMITONES enrichment scores differ greatly from gene ranks based on singleCellHaystack adjusted *P*-values. Genes that rank highly according to singleCellHaystack often also do so according to SEMITONES, but several well-known marker genes that ranked highly by SEMITONES are assigned low ranks by singleCellHaystack. (**B**) Several genes ranked lowly by SEMITONES are assigned rank 1, i.e. the *q*-value is 0, by Seurat v3’s default Wilcoxon rank-sum test. Contrastingly, only few high ranking genes according to SEMITONES (rank < 10) rank lowly according to Seurat v3 (rank > 100). (**C**) Example expression profiles of genes that are only identified by SEMITONES and Seurat v3 and not singleCellHaystack (left column), that are only identified by Seurat v3 (middle column), and that are only identified by SEMITONES (right column). (**D**) Comparison of the performance and runtime between SEMITONES and several alternative marker identification methods on simulated scRNA-seq data. Due to runtime constraints, the median AUC of only 12 of the simulated datasets containing 10 000 cells is presented for the unfiltered MAST and Wilcoxon methods. Likewise, the median runtime is given for just 8 and 6 10 000-cell simulations for MAST and Wilcoxon, respectively. In all other cases, the median over all 20 simulations is reported. In the AUROC plot, the grey dashed line indicates the expected AUROC for a random classifier. In the runtime plot, we provide the runtime with (triangle markers) and without (circle markers) significance testing.

In the Discussion section, the authors also made the follow adjustment. Instead of ‘Additionally, we use simulated scRNA-seq data to verify the accuracy of SEMITONES and establish its superior performance over alternative feature identification methods for single-cell transcriptomic data.’ This fragment should read: ‘Additionally, we use simulated scRNA-seq data to verify the accuracy of SEMITONES, to showcase that SEMITONES achieves comparable performance to differential testing even when differential testing methods are provided with ground truth cluster labels, and establish its superior performance over alternative clustering-independent feature identification methods for single-cell transcriptomic data.’

This error has been corrected online.

